# Depiction of immune heterogeneity of peripheral blood from patients with type II diabetic nephropathy based on mass cytometry

**DOI:** 10.3389/fendo.2022.1018608

**Published:** 2023-01-06

**Authors:** Juan Jin, Longqiang Wang, Yongjun Liu, Wenfang He, Danna Zheng, Yinhua Ni, Qiang He

**Affiliations:** ^1^ Urology & Nephrology Center, Department of Nephrology, Zhejiang Provincial People’s Hospital, Affiliated People’s Hospital, Hangzhou Medical College, Hangzhou, Zhejiang, China; ^2^ Department of Thyroid and Breast Surgery, The Central Hospital of Wuhan, Tongji Medical College, Huazhong University of Science and Technology, Wuhan, China; ^3^ College of Biotechnology and Bioengineering, Zhejiang University of Technology, Hangzhou, China; ^4^ Department of Nephrology, The First Affiliated Hospital of Zhejiang Chinese Medical University (Zhejiang Provincial Hospital of Traditional Chinese Medicine), Hangzhou, Zhejiang, China

**Keywords:** high-dimensional mass cytometry, diabetic nephropathy, immune disorder, peripheral blood mononuclear cell (PBMC), type II diabetes mellitus

## Abstract

Diabetic nephropathy (DN) is the most prominent cause of chronic kidney disease and end-stage renal failure. However, the pathophysiology of DN, especially the risk factors for early onset remains elusive. Increasing evidence has revealed the role of the innate immune system in developing DN, but relatively little is known about early immunological change that proceeds from overt DN. Herein, this work aims to investigate the immune-driven pathogenesis of DN using mass cytometry (CyTOF). The peripheral blood mononuclear lymphocytes (PBMC) from 6 patients with early-stage nephropathy and 7 type II diabetes patients without nephropathy were employed in the CyTOF test. A panel that contains 38 lineage markers was designed to monitor immune protein levels in PBMC. The unsupervised clustering analysis was performed to profile the proportion of individual cells. t-Distributed Stochastic Neighbor Embedding (t-SNE) was used to visualize the differences in DN patients’ immune phenotypes. Comprehensive immune profiling revealed substantial immune system alterations in the early onset of DN, including the significant decline of B cells and the marked increase of monocytes. The level of CXCR3 was dramatically reduced in the different immune cellular subsets. The CyTOF data classified the fine-grained differential immune cell subsets in the early stage of DN. Innovatively, we identified several significant changed T cells, B cell, and monocyte subgroups in the early-stage DN associated with several potential biomarkers for developing DN, such as CTLA-4, CXCR3, PD-1, CD39, CCR4, and HLA-DR. Correlation analysis further demonstrated the robust relationship between above immune cell biomarkers and clinical parameters in the DN patients. Therefore, we provided a convincible view of understanding the immune-driven early pathogenesis of DN. Our findings exhibited that patients with DN are more susceptible to immune system disorders. The classification of fine-grained immune cell subsets in this present research might provide novel targets for the immunotherapy of DN.

## Introduction

With the rapid prevalence of diabetes mellitus (DM) worldwide, the corresponding morbidity, mortality, and medical cost are staggering. The 10^th^ edition of the International Diabetes Federation (IDF) recently reports that 537 million adults aged 20 to 79 will live with diabetes in 2021. This number is predicted to rise to 783 million in 2045 (1). Chronic hyperglycemia results in severe clinical complications, including diabetic retinopathy, diabetic cardiovascular diseases, and diabetic nephropathy (DN). DN is the most common renal damage affecting approximately 40% of type 1 and type 2 patients with DM (T2DM) (2). The early morphological change of renal impairment is generally detected by nephromegaly and a modified Doppler, but the evidence of proteinuria (≥ 30 and < 300 mg/day) and a significant decrease in glomerular filtration rate (GFR) were defined as the best ascertained clinical characteristics of early-stage of DN (3). However, glomerular damage arises when albumin appears in the urine; urinary albumin cannot accurately identify the risk of developing DN. Therefore, novel urinary biomarkers and related mechanisms are recently reported, such as oxidative stress, inflammation, and renin-angiotensin-aldosterone system (RAAS) activation (4). Motawi et al. suggested three novel potential biomarkers for early detection of DN in T2DM patients’ serum: neutrophil gelatinase-associated lipocalin (NGAL), beta-trace protein (beta TP), and microRNA-130b (miR-130b) (5). However, these biomarkers are neither calibrated nor commonly available in clinical applications, so more reliable biomarkers must be studied urgently.

Systematic and local inflammation in the kidney is defined as the leading cause of the pathogenesis of DN. DN is associated with metabolic and hemodynamic disorders, which recruit many white blood cells in the kidney. Then a variety of pro-inflammatory cytokines and chemokines secreted by leukocytes may be centralized into the kidney directly to motivate the innate immune system (4). In addition, the adaptive immune system activation is mainly induced by CD4^+^ T cells-differentiated subsets, such as T-helper 1(Th1), Th2, Th17, and Treg, which produce excessive inflammatory cytokines (i.e., interferon-γ (IFN-γ), interleukin-4 (IL-4) and IL-17), and suppress T cells to maintain immune balance (6). It has been well adapted that T cell-mediated immune activation showed an essential role in the pathogenesis of type 1 DM by developing insulitis (7). By contrast, T2DM is a nonautoimmune form mainly characterized by insulin resistance. Currently, there is little evidence regarding the substantial role of T cells in the pathogenesis of T2DM and related DN. Furthermore, C-X-C motif chemokine receptor 3 (CXCR3) is highly expressed on Th1-type CD4^+^ T cells and cytotoxic lymphocytes CD8^+^ cells, which drives entry of activated T cells into inflamed tissues during T cell priming and recall immune responses (8). One clinical study demonstrated that CXCL10-CXCR3 interaction contributed to the selective disruption of pancreatic β cells in the progress of T1DM (9). However, the role of CXCR3 in the pathogenesis of T2DM and complicated DN remains elusive.

Traditional fluorescence cytometry is still a mainstream approach to detecting and analyzing the immune system on a cellular level. Still, the available fluorescence flux is a low and frequent overlap of emission spectra of different fluorescent labels (10). Mass cytometry (cytometry by time-of-flight, CyTOF) is recently developed as a novel and emerging technology for multiparameter single cell analysis, which adopts heavy metal ions as antibody labels and thus approximately no background (10). The precision of distinct mass resolution overcomes the low-fluorescence flux in flow cytometry, which can combine around 40 tags in one sample (11). Therefore, the present work employed CyTOF for the peripheral blood mononuclear lymphocytes (PBMC) samples from 6 patients with early-stage nephropathy and 7 type II diabetes patients without nephropathy to detect the characterizations of a significant number of immune cells between T2D patients with or without DN. In addition, our study provides evidence and potential immune targets for the immune-driven pathogenesis of T2DM-associated DN.

## Materials and methods

### Subjects and sample preparation

Six T2DM patients with early stage of DN (T2D-DN) and seven T2DM patients without DN (T2D) were enrolled in the present study. The clinical characteristics of the patients are summarized in **Table 1**. The subjects were employed from the Affiliated Lin’an People’s Hospital and age-matched without any other diseases. No drugs were taken within two weeks before the sample collection. The heparinized peripheral blood samples were collected for subsequent analysis in the CyTOF test. Peripheral blood mononuclear cells (PBMCs) were isolated from EDTA-anticoagulated blood samples on a Ficoll-Histopaque density gradient following the manufacturer’s instructions. The Affiliated Lin’an Hospital Ethic Committee approved the present study, and informed consent was obtained from all participants. We obtained informed consent from the subjects who joined the current protocol.

**Table 1:** Clinical characteristics of 6 T2DM patients with DN and 7 T2DM patients without DN.

Patients’ ID	DN	Age	Gender	BMI	TP (g/l)	HAS (g/l)	BUN (mM/l)	CRE (μM/l)	FPG (mM/l)	GFR (ml/min)	HbA1c (%)	PRO	OB	LEU	UTA/CRE (mg/mM)	UTF/CRE	UIG/CRE	Stage of CKD	Hypertension
Patient 1	YES	65	female	19.9	37.6	17.3↓	4.2	77.3	6.28	77.07↓	10.9	++	+	++	830↑	48.2↑	92.3↑	G2	YES
Patient 2	YES	75	male	30.7↑	53.8	28.8↓	8.25↑	117.9↑	6.53	56.91↓	8.8	+++	++	–	854.6↑	62.8↑	115.6↑	G3a	YES
Patient 3	YES	47	male	20.8	56.6	29.4↓	12.18↑	173.8↑	3.56	38.14↓	5.9	+++	+	–	608.7↑	38.6↑	91↑	G3b	YES
Patient 4	YES	50	female	24.2	66.3	27.6↓	13.97↑	268.6↑	5.91	17.54↓	7.7	++	+	Low	273.7↑	17.6↑	74.3↑	G4	YES
Patient 5	YES	64	male	25.9↑	55.9	24.1↓	3.39	76.7	9.11	99.04↓	8.8	+++	++	–	711.9↑	53.7↑	87↑	G1	YES
Patient 6	YES	78	female	19.2	74.3	39	18.51↑	264.6↑	5.45	20.89↓	12.4	++	++	–	63.3↑	3.4↑	11.4↑	G4	YES
Patient 7	NO	66	female	22.8	63.9	38.2	4.68	62.6	8.26	100.25	11.2	–	–	–	2.1	<2.3/10.2	0.7	G1	NO
Patient 8	NO	55	male	19.1	53.5	33.9	4.5	77.7	4.38	100.82	8.4	–	–	–	<10.9/12.75	<2.3/12.75	<3.5/12.75	G1	NO
Patient 9	NO	53	male	24.5	67.3	40.5	4.87	56.6	7.78	150.62	11.3	–	–	–	1.8	<2.3/8.2	0.9	G1	NO
Patient 10	NO	62	male	25.2	71.9	40.4	5.59	67.3	5.16	117.39	8.8	–	–	–	0.7	<2.3/19.43	0.5	G1	NO
Patient 11	NO	44	male	25.6↑	55.3	33.8	4.51	62.6	11.75	137.22	11.6	–	–	–	1.8	<2.3/11.31	0.6	G1	NO
Patient 12	NO	57	male	23.3	70.6	40.3	4.19	73.1	7.33	107.3	10.8	–	–	–	1.05	0.07	1.03	G1	YES
Patient 13	NO	48	female	24.3	61.2	33.2	4.31	78.8	4.89	100.49	9.2	–	–	–	<8/10.87	0.38	<4/10.87	G1	NO

Arrows indicate an increase (↑) or decrease (↓) in the T2DM patients with or without DN.

DN, diabetic nephropathy; BMI, body mass index; TP, total protein; HAS, albumin; BUN, urea nitrogen; CRE, creatinine; FPG, fasting plasma glucose; GFR, glomerular filtration rate; HbA1c, hemoglobin A1C; PRO, urine protein; OB, urine occult blood; LEU, urine leukocyte; UTA/CRE, urine trace albumin/creatinine; UTF/CRE, urine transferrin/creatinine; UIG/CRE, urine immunoglobulin/creatinine; Stage of CKD, stage of chronic kidney disease. "-", "Low", "+", "++" and "+++" : "-" means negative, "Low" means low amount of PRO, OB and LEU, "+" represents the amount of PRO, OB and LEU, more "+" means higher amount of PRO, OB and LEU.

### PBMC staining for CyTOF

PBMCs were suspended in flow cytometry (FACS) Buffer (Sigma, USA) with 250 nM monoisotopic cisplatin reagents Cell-ID Cisplatin-194Pt (Fluidigm, San Francisco, CA) for viability staining. Fc-receptor blocking solution (BioLegend, San Diego, CA) was used for cell blocking, followed by incubation with an extracellular antibody cocktail for 30 minutes on ice. Then the cells were fixed and permeabilized using intercalation solution (Fluidigm) and stained with the intracellular markers for 30 minutes on ice. As described previously, the mass-tag cell barcoded (MCB) were used to eliminate variability between antibody staining samples and instrument sensitivity (12). Finally, the cells were resuspended in deionized water with 20% (V/V) EQ beads (Fluidigm) and filtered into cell strainer cap tubes.

### Mass cytometry (CyTOF)

**Table S1** contains all the 38 metal-conjugated antibodies with specifications used in CyTOF experiments. EQ beads (Fluidigm) were used as a loading control. All data were produced on a Helio3 CyTOF Mass Cytometer (Fluidigm). Mass cytometry data files were normalized using the bead-based Normalizer (13). The output FCS files were randomized and homogenized with the EQTM Four Element Calibration beads against the entire run, per the manufacturer’s recommendations (14). CyTOF analysis was performed by PLTTech Inc. (Hangzhou, China) as described previously (15).

### Statistical analysis

All 38 immune cell markers were applied for clustering and visualization. X-shift algorithm was used to cluster cells. Twenty thousand cells were selected randomly for visualizing by t-Distributed Stochastic Neighbor Embedding (t-SNE) using the R package cytofkit (16). Immune subset cells were defined by the median values of specific expression markers on Hierarchical clustering (17). Two-group comparisons were assessed using unpaired Student’s t-tests. These tests were justified based on an assessment of normality and variance of the data distribution. *P*-values < 0.05 or 0.01 were considered statistically significant.

## Results

### Peripheral immunity signature traits in the patients with early onset of DN

**Figure 1:**
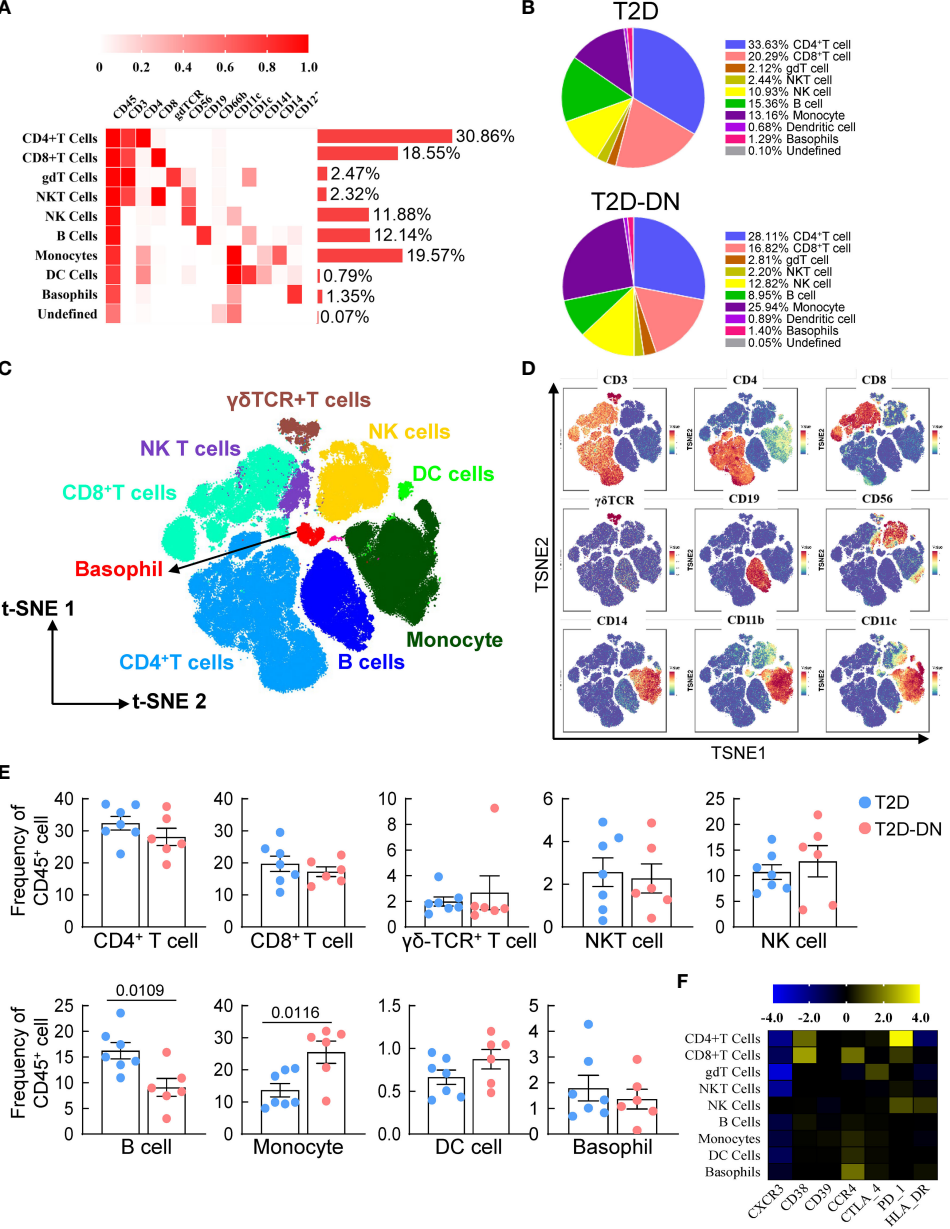
Peripheral immunity signature traits in the early-stage diabetic nephropathy patients. **(A)** Heatmap analysis for the overall proportions of major immune cell subsets in diabetic nephropathy **(B)** Pie chart characterizing the differences of major immune cell subsets proportions between T2D-DN and T2D patients. **(C)** Distributions of major immune cell subsets **(D)** The key immune cell markers for immune cell subsets are analyzed by t-SNE algorithm. **(E)** Statistical frequency differences of immune cell subsets between T2D-DN and T2D patients. **(F)** Heatmap analysis for the expressions of functional immune cell markers in the measurable immune cell subsets. Data are expressed as means ± SEM, n = 6 in T2D-DN group and n = 7 in T2D group.

Six T2DM subjects with early stages of DN (T2D-DN) and seven T2DM issues without DN (T2D) participated in the CyTOF test to investigate the dynamic profiling of the peripheral immune microenvironment. We selected CD45^+^CD66b^-^ -marked PBMCs and performed hierarchical clustering analysis in the 13 samples from patients *via* the X-shift algorithm. Nine major immune cell subsets characterized by different signaling antibodies were defined in **Figures 1A, B**. T cells and monocytes were the central and peripheral immune cells, which account for 49.41% and 19.57%, respectively (**Figure 1B**). CD4-positive T cells were the most abundant immune cell subsets accounting for 30.86%, and CD8^+^, γδ T cell, NKT cell, NK cell, and B cell were respectively accounting for 18.55%, 2.47%, 2.32%, 11.88% and 12.14% (**Figure 1B**). Dendritic cells (DC) and basophils were small immune cell subsets (**Figure 1B**). Following t-SNE analysis was performed to visualize the different proportions of immune cell subsets between the T2D-DN and T2D groups (**Figures 1C, D**). The ratio of B cells was significantly decreased in the T2D-DN group. At the same time, the proportion of monocytes was markedly increased compared with the T2D group (**Figure 1E**). However, other defined immune cell subsets did not significantly change between T2D-DN and T2D patients (**Figure 1E**). Notably, the level of CXCR3 exhibited a considerable decrease in the defined immune cell subsets in the T2D-DN group (**Figure 1F**). In addition, C-C motif chemokine receptor 4 (CCR4) and CD38 were upregulated in the CD8^+^ T cells. The programmed death-1 (PD-1) dramatically increased in the CD4^+^ T cells (**Figure 1F**).

### The immunological specificity of T cell subsets in the T2D-DN patients

**Figure 2:**
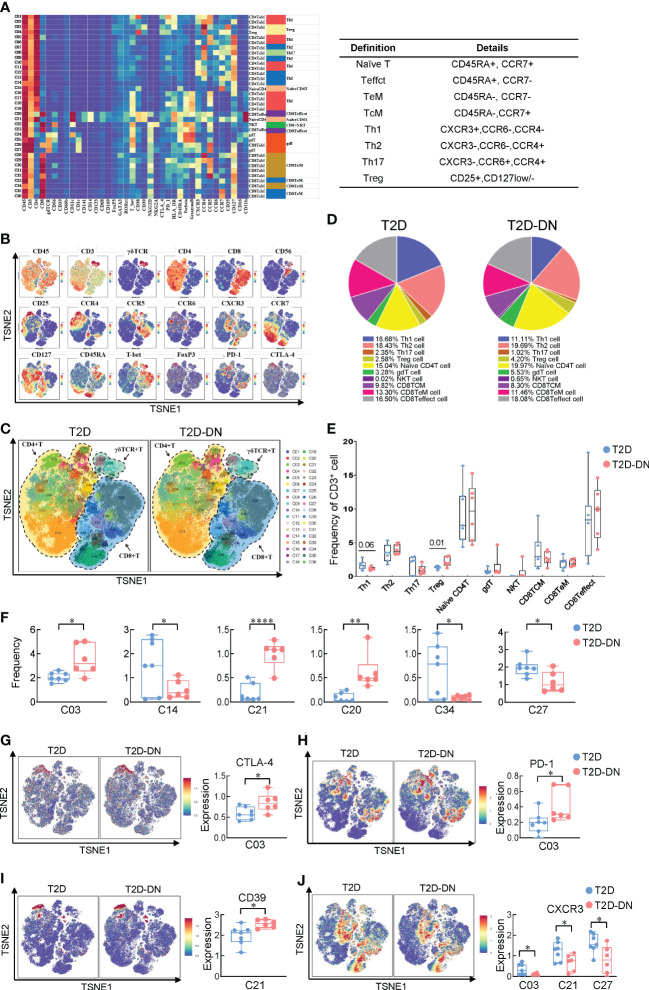
Identification of immune heterogeneity of T cell subsets. **(A)** Heatmap exhibiting the expressions of 38 immune cell markers in the T cell subsets (left) and 8 marked definitions of T cell subsets (right). **(B)** Distributions of functional traits are expressed in the different T cell subsets. **(C)** Island maps exhibit the measurable T cell subsets distribution. **(D)** Pie chart characterizing the differences of primary T cell subsets proportions between T2D-DN and T2D patients. **(E)** Statistical frequency differences of T cell subsets between T2D-DN and T2D patients. **(F)** Significantly changed T cell subsets between T2D-DN and T2D patients. **(G)** CTLA-4, **(H)** PD-1, **(I)** CD39, and **(J)** CXCR3 in the specific T cell subsets between T2D-DN and T2D patients. Data are expressed as means ± SEM, n = 6 in T2D-DN group and n = 7 in T2D group., *P < 0.05, **P < 0.01, ****P < 0.0001 vs. T2D group.

To identify the substantial role of T cells in the pathogenesis and progress of DN, we performed a single-linkage clustering analysis focusing on CD45^+^CD19^-^CD3^+^-labeled T cell subsets between T2D-DN and T2D groups (**Figures 2A, B**). Eight definitions of CD4^+^ and CD8^+^ T cell subsets consisted of The CD45RA^+^CCR7^+^-Naïve T cell, CD45RA^+^CCR7^–^Teffct cell, CD45RA^-^CCR7^–^TeM cell, CD45RA^-^CCR7^+^-TcM cell, CXCR3^+^CCR6^-^CCR4^–^Th1 cell, CXCR3^-^CCR6^-^CCR4^+^-Th2 cell, CXCR3^-^CCR6^+^CCR4^+^-Th17 cell, and CD25^+^CD127^low–^Treg cell (**Figure 2A**).

Generally, the proportion of the Th1 cell subset was significantly decreased while the proportion of Treg cell subsets was markedly increased in the T2D-DN group, compared with those in the T2D group (**Figures 2C–E**). However, other T cell immune subsets did not show the observed change between T2D-DN and T2D patients (**Figure 2E**). Specifically, the proportion of C03 (CD4TeM cell), C21 (NaiveCD4 T cell), and C20 (CD8Teffect cell) were significantly increased in the T2D-DN group. In contrast, the proportion of C14 (CD4TeM cell), C34 (CD8TeM cell), and C27 (gdT cell) were dramatically decreased in the T2D-DN group when compared with those in the T2D group (**Figure 2F**). Those results implied that the innate immune system was activated intensely during the early onset of kidney disorder in T2DM patients. Visualized results showed that the subgroup of C03 subset significantly increased in the T2D-DN group associated with the upregulation of immunological inhibitory receptors, such as cytotoxic t-lymphocyte antigen 4 (CTLA-4) and program death-1 (PD-1) (**Figures 2G, H**). However, the level of CXCR3 showed a significant decrease in C03 subset, C21 subset, and C27 subset in the T2D-DN group, while the CD39 expression was significantly increased in C21 NaiveCD4 T cell of T2D-DN patients, compared with those in T2D group (**Figures 2I, J**).

### The potential role of B cell subsets in the T2D-DN patients

**Figure 3:**
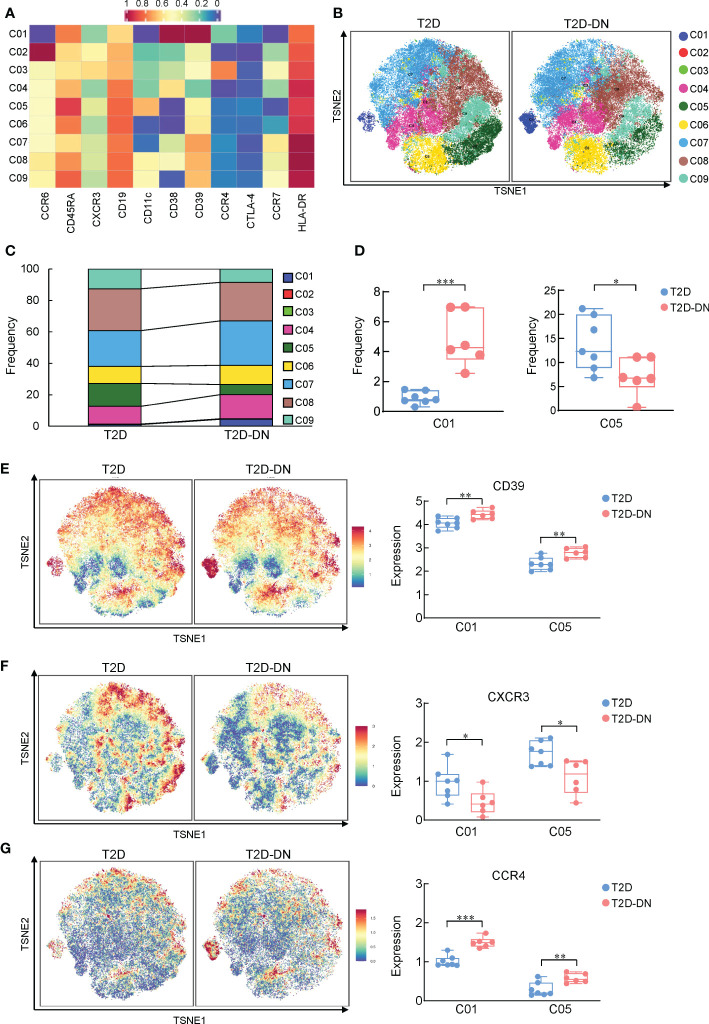
Depiction of features of B cell subsets between T2D-DN and T2D patients. **(A)** Heatmap exhibiting the expressions of immune cell markers in the B cell subsets. **(B)** Island maps display the measurable B cell subsets distribution. **(C)** Statistical frequency differences of B cell subsets between T2D-DN and T2D patients. **(D)** Significantly changed B cell subsets between T2D-DN and T2D patients. **(E)** CD39, **(F)** CXCR3 and **(G)** CCR4 in the specific B cell subsets between T2D-DN and T2 patients. Data are expressed as means ± SEM, n = 6 in T2D-DN group and n = 7 in T2D group., *P < 0.05, **P < 0.01, ***P < 0.001 vs. T2D group.

Since B cells also act as an essential player in immune-driven metabolic disorders, we also analyzed the different CD45^+^CD19^+^CD3^-^ -labeled B cell subsets between the T2D-DN and T2D groups. CyTOF data defined 9 B cell subgroups based on the clustering analysis (**Figure 3A**). The distribution and frequency proportions of these measurable subsets were shown in the t-SNE graph (**Figures 3B, C**). Interestingly, we found that the proportion of cluster 1 (C01) exhibited a significant increase in the T2D-DN group. In contrast, the proportion of cluster 5 (C05) decreased significantly in the T2D-DN patients compared with those in the T2D patients without DN (**Figure 3D**). C01 was defined as a kind of B cell subsets that expressed mostly CD38^+^CD39^+^ but did not express CCR6 and CD1c, whereas C05 mainly said CCR6^+^CD1c^+^CD39^+^ but did not express CD38 (**Figure 3B**). Notably, the expression of CD39 and CCR4 showed a significant increase both in C01 B cell subset and C05 subset in the T2D-DN group compared with those in the T2D patients without DN (**Figures 3E, G**). In addition, the level of CXCR3 was significantly decreased both in the C01 and C05 in the T2D-DN patients (**Figure 3F**), which was consistent with previously mentioned results in T cell subgroups (**Figure 2J**).

### The immunological heterogeneity of myeloid cells in the T2D-DN patients

**Figure 4:**
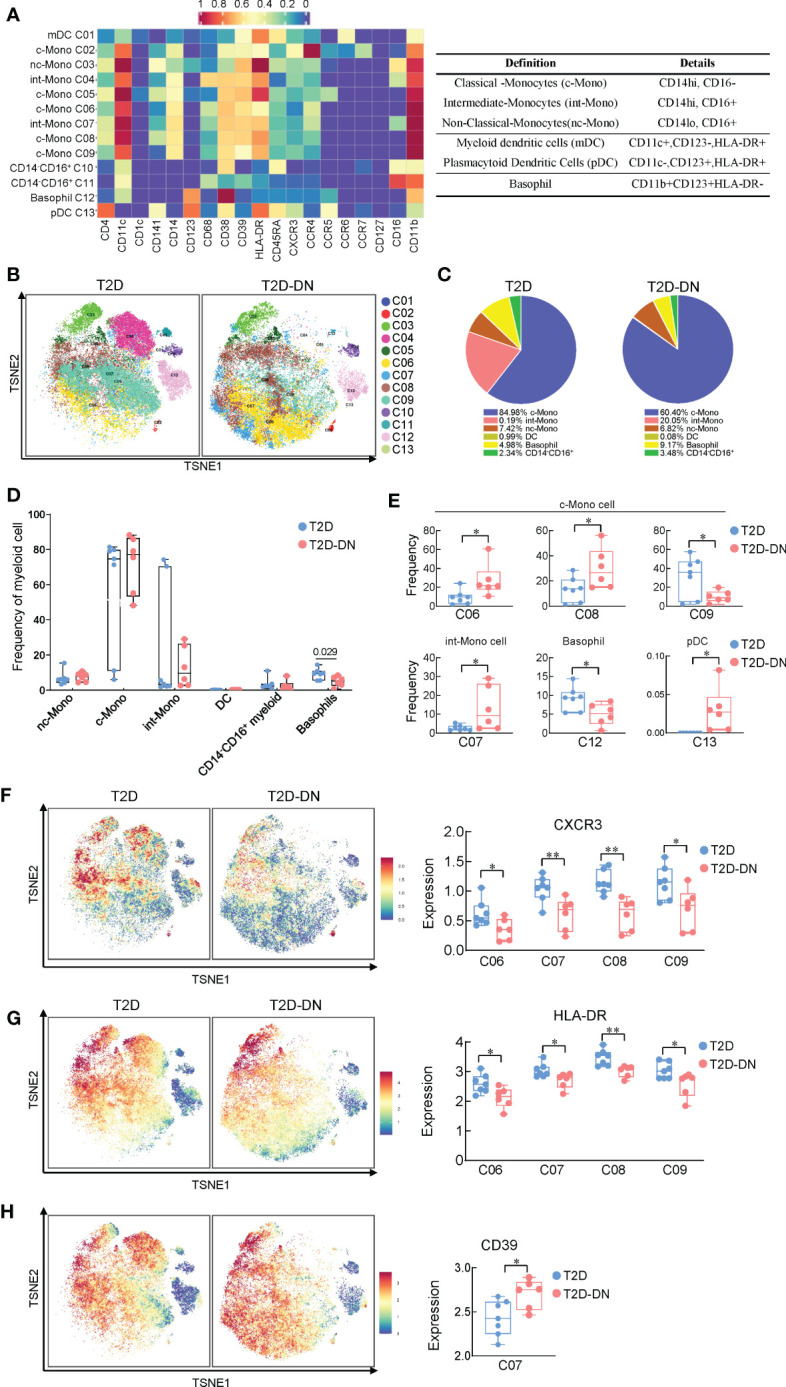
Immunological heterogeneity of myeloid cell subsets between T2D-DN and T2D patients. **(A)** Heatmap exhibiting the expressions of immune cell markers in the myeloid cell subsets (left) and six marked definitions of myeloid cell subsets (right). **(B)** Island maps exhibit the measurable myeloid cell subsets distribution. **(C)** Pie chart characterizing the differences of significant myeloid cell subsets proportions between T2D-DN and T2D patients. **(D)** Statistical frequency differences of myeloid cell subsets between T2D-DN and T2D patients. **(E)** Significant changed myeloid cell subsets between T2D-DN and T2D patients. **(F-H)** The level of CXCR3 **(F)**, HLA-DR **(G)**, and CD39 **(H)** in the specific myeloid cell subsets between T2D-DN and T2D patients. Data are expressed as means ± SEM, n = 6 in T2D-DN group and n = 7 in T2D group., *P < 0.05, **P < 0.01 vs. T2D group.

We next compared the different expressions of myeloid cells between the T2D-DN and T2D groups *via* the characteristic markers of CD14, CD11c, CD11b, CD123, HLA-DR, and CD16 (**Figures 4A, B**). Most myeloid cells did not exert significant change between T2D-DN and T2D groups, but only the proportion of basophil showed a considerable decrease in the T2D-DN patients (**Figures 4C, D**). Specifically, the ratios of C06 (classical-monocytes), C08 (classical-monocytes), C07 (intermediate-monocytes), and C13 (plasmacytoid dendritic cells) were significantly increased in the T2D-DN group (**Figure 4E**). In contrast, the proportions of C09 (classical-monocytes) and C12 (basophil) were markedly decreased in the T2D-DN group compared with those in T2D patients without DN (**Figure 4E**). Interestingly, the level of CXCR3 and human leukocyte antigen-DR isotype (HLA-DR) were both significantly decreased among C06, C07, C08, and C09 subgroups in the T2D-DN group (**Figures 4F, G**). In contrast, the CD39 expression was significantly increased in the T2D-DN patients compared with the T2D group (**Figure 4H**).

### Correlations between identified immune populations and renal clinical parameters in the T2D-DN patients

**Figure 5:**
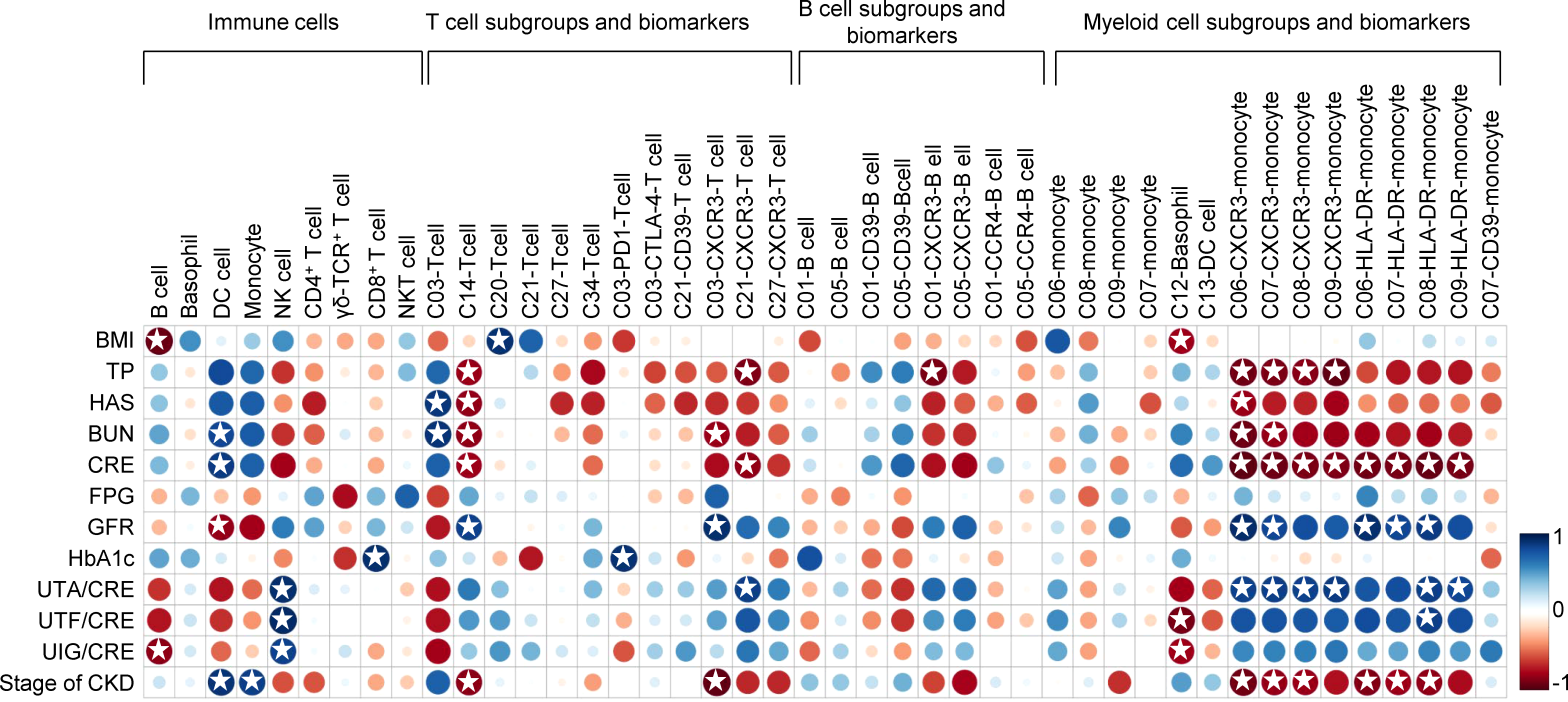
Correlation analysis between identified immune populations and renal clinical parameters as well as diabetic markers in the T2D-DN patients. Rows correspond to renal clinical parameters and diabetic markers, and columns correspond to the specific immune cell subsets and biomarkers. Blue and red colors denote positive and negative associations, respectively. The intensity of the colors represents the degree of association between the immune populations and clinical parameters assessed by Pearson correlation analysis. Stars means *P* < 0.05.

Pearson correlation analysis was performed between above changed immune cell subgroups and specific renal clinical parameters in the T2D-DN group, including albumin, urea nitrogen, creatinine, glomerular filtration rate, as well as several diabetic markers, such as body mass index (BMI), blood glucose and hemoglobin A1C (HbA1c) level (**Figure 5**). In general, these identified immune cell subsets and related biomarkers were significantly correlated with the clinical diabetic nephropathy-related traits in the T2D-DN subjects. Firstly, the decreased ratio of B cells was significantly and negatively correlated with the proportion of urine immunoglobulin and creatinine (UIG/CRE), which was regarded as a significant diagnostic marker for DN. Also, the increased level of monocytes in the T2D-DN group exhibited a significant positive correlation with the stage of chronic kidney disease (CKD). Notably, the increased proportion of C03 (CD4TeM cell) in the T2D-DN group showed a marked positive correlation with the level of albumin (HAS) and urea nitrogen (BUN). Conversely, the decreased proportion of C14, C27, and C34 T cell subgroups negatively correlated with the clinical DN markers, such as total protein (TP), HAS, BUN, and CRE, in which the C14 subset showed a significant association with the above parameters.

Moreover, the decreased level of CXCR3 in different identified T cell subsets (i.e., C03, C21, and C27), B cell subsets (i.e., C01 and C05), and myeloid cell subsets (i.e., C06, C07, C08, and C09) consistently showed a negative correlation with the level of TP, HAS, BUN, CRE, while positively related with the ratio of urine trace albumin and creatinine (UTA/CRE), urine transferrin and creatinine (UTF/CRE), and UIG/CRE in the T2D-DN subjects. In addition, a similar correlation trend was also exhibited on the level of HLA-DR in the different monocyte subgroups, including C06, C07, C08, and C09 subpopulations. On the other hand, the decreased proportions of B cells and the C12 basophil subset showed a significant negative correlation with BMI. In contrast, the increased proportions of the C20 T cell subset were markedly positively related to BMI in the T2D-DN group. In addition, the increased level of PD-1 in the C03 T cell subgroup exhibited a significant positive correlation with HbA1c level in the T2D-DN subjects.

## Discussion

Diabetic nephropathy leads to the progression of end-stage kidney disease (ESKD). Clinically, severe cardiovascular disease and vascular problems are the most common causes of poor prognosis of diabetes with ESRD (18). At the same time, since most patients with late stages of DN should receive renal transplantation and become more susceptible to infections, it is essential to use immunosuppressive regimens after transplantation (19). Therefore, the complicated drug intake and interventions in the late stage of DN patients significantly change the immune microenvironment, which could not reflect the immune cell alternations during the progression of DN. Still, most pioneering studies were based on the characteristics of late DN, and relatively little is known about early pathological change that proceeds from overt DN. Increasing evidence is emerging supporting the role of the innate immune system and circulating inflammatory cytokines in the development of DN (20). In 2019, Wilson et al. found an approximate seven- to eight-fold increase in the leukocytes of early-stage DN patients compared with control using unbiased single-nucleus RNA sequencing (21). However, the complex mechanism of immune-driven kidney complications in T2D pathology remains elusive. In the current study, we described an immune-driven pathophysiological change of early-stage DN in T2D patients *via* CyTOF analysis. Further, we identified several potential immune biomarkers for the early onset of DN.

It is widely accepted that T cell abnormal expression is commonly observed in the development of DN. Remarkably, the circulating activated T lymphocyte levels were dramatically higher in type 1 diabetic patients with proteinuria than in non-proteinuria patients (22). However, we did not find a significant change in T cell populations between T2D-DN and T2D patients (**Figures 1B, D**). It is well recognized that Th1 cells mainly secrete IFN-γ to produce the proinflammatory responses and present antigens to T lymphocytes (23). One previous study reported an increase of proinflammatory cytokines, Th1 cytokines, and chemokines, but not Th2 cytokines, in the plasma and urine of patients with DN as compared to T2DM patients without nephropathy, followed by a positive correlation between plasma IFN-*γ*, proteinuria, and GFR in the T2DM patients with DN (24). Consistently, the proportion of Th1 but not Th2 cells was significantly elevated in the T2D-DN patients as compared with T2D subjects (**Figure 2E**). We identified six T cell subgroups significantly upregulated or downregulated in the T2D-DN patients, which might be the potential T cell characteristic groups that occurred in the early stage of DN (**Figure 2F**). In addition, regulatory T cells (Tregs) were postulated to regulate diabetes progression by suppressing the activation of effector T cells and exerting anti-inflammatory impacts. Still, the substantial role of Tregs in the pathogenesis of DN seems to remain controversial. Eller et al. proved that adoptive transfer of CD4^+^FoxP3^+^ Tregs significantly increased in obese T2D patients with DN and improved insulin sensitivity (25). Conversely, Zhang et al. reported markedly decreased CD4+CD25+Foxp3+ Treg cells in T2D-DN patients, followed by a negative correlation between urinary albumin creatinine ratio and the proportion of Treg cells (26). Our present data showed that the proportion of Treg cells was significantly increased in the early onset of DN. In addition, our data showed that different T cell subsets showed robust correlation with clinical parameters of DN in the T2D patients with DN. Further studies are required to identify the difference in Treg cell populations between the early- and end-stage T2D-DN patients.

Overall, six T2D-DN subjects and seven T2D subjects exhibited vastly different peripheral immunity signature traits in the PBMC, mainly embodied in the marked downregulation of B cells and upregulation of monocytes and the T2D-DN patients (**Figure 1**). There is little evidence to prove the direct role of B cells in the progression of DN. One clinical study revealed that circulating regulatory B cells were significantly lower in DN patients than DM control group (27). Also, we identified CD38^+^CD39^+^ CCR6^-^CD1c^-^ C01 subgroup and CCR6^+^CD1c^+^CD39^+^CD38^-^ C05 subgroup as the significant change and potential B cell subsets in the T2D-DN patients. Also, CCR4 was significantly upregulated in the B cell subsets, which might be the early diagnostic B cell biomarker during the progression of DN. Increasing studies have demonstrated that monocytes or macrophages infiltration in the kidney was the representative event of the final diabetic renal damage, triggered by the release of proteolytic enzymes, oxygen radicals, and pro-inflammatory cytokines (28). One recent study reported that serum and urinary monocyte chemoattractant protein-1 (MCP-1) levels were elevated in the early-stage DN patients, which acted as a potent chemokine to recruit monocytes (29). Although we did not find a significant change in total monocyte populations, only basophils decreased markedly in the T2D patients with early DN. CyTOF analysis demonstrated classical monocytes C06, C07, and C08 subgroups significantly increased, but C09 subgroups decreased dramatically in the DN patients (**Figures 4D, E**). Collectively, our present data further support that monocyte infiltration and B cell downregulation might be the convincing markers during the early onset of DN. However, to identify the role of immune-driven inflammatory response in the progression of DN, further research should focus on the level of monocyte-secreted cytokines and chemokines in T2D patients with nephropathy and without nephropathy.

Notably, we identified several potential biomarkers that occurred in the early onset of DN in T2D patients. For example, cytotoxic T-lymphocyte-associated antigen-4 (CTLA-4) significantly increased in the C03 T cell subset of T2D-DN patients, which only expressed on activated Th cells and negatively regulated T-cell response for the inflammatory reaction (30). Jacob et al. demonstrated a significantly increased frequency of CTLA-4-318C/T genotype in IgA nephropathy patients (31). Thus, our present findings suggested a possible biomarker for CTLA-4 in Th cells in DN. In addition, one previous clinical study has reported a significant increase in the circulating CD4^+^CXCR5^+^PD-1^+^, PD-1^+^CD154^+^, PD-1^+^-IL-17^+^-T follicular helper (Tfh) cell counts in the PBMCs of a total of 23 DN patients (32). In parallel, we observed a significant increase of PD-1 in C03 T cell subsets in the T2D-DN subjects compared with that in T2D control patients, which implied the potential role of PD-1 in diagnosing and intervening early DN. However, another study reported a case of acute kidney injury in 15 patients who received anti–PD-1 therapy (33). Thus, the therapeutic role of PD-1 in kidney diseases is required to be further elucidated. Human leukocyte antigen (HLA) has been robustly replicated as a genetic risk factor in type 1 diabetes (34), but its concrete role in T2DM and related nephropathy remains elusive. Our data found a significant decrease of HLA-DR in the monocyte subgroups as well as the robust correlation with clinical diagnostic markers in the T2D patients with early DN, which provided a potential biomarker for immune-driven diabetic nephropathy.

One of the most exciting findings in the present study was the marked downregulation of CXCR3 in the measured immune cells, particularly in the T cells, B cells, and monocytes. High expressions of chemokines and their receptors in the infiltrating immune cells are recognized as the characteristic events in the progression of DN and CXCR3, and its ligands, CXCL9, CXCL10, and CXCL10 are the well-known risk factors for inflammatory-based kidney diseases (35). In human glomerulonephritis, CXCR3 is mainly expressed in the interstitial infiltrating T cells, and their number correlates with renal function, proteinuria, and percentage of globally sclerosed glomeruli (36). There is little evidence for the direct role of CXCR3 in the development of DN, and a previous study has reported a significantly elevated urinary level of CXCL9 in patients with primary renal disease and increased urinary level albumin excretion rate (37). Therefore, CXCR3 might be selectively liganded with CXCL9 and might be the candidate that plays a role in the immune cell infiltration into the kidney complications during the progression of T2DM. In addition, correlation analysis data showed that significantly altered levels of CXCR3 in different identified T cell subsets, B cell subsets, and myeloid cell subsets dramatically exhibit strong correlations with the clinical diagnostic targets in the T2D-DN subjects (**Figure 5**). Our data supported CXCR3 as an early biomarker in the DN. In addition, one clinical study reported an increase of CD4^+^Foxp3^+^CD39^+^ Treg cells in PBMCs from overweight T2DM patients associated with hyperglycemia (38), and we observed an elevated level of CD39 among T cells, B cells, and monocytes. Thus, we believed that CD39 also exerted potential in the early verification of DN. Glucose metabolism and related biochemical criteria should be considered to determine the substantial role of CD39 in further research.

In summary, our present study revealed substantial immune cell alterations during the early onset of DN *via* CyTOF analysis, including the significant decline of B cells and the marked increase of monocytes. Th1 cell subsets and Treg cells exhibited a considerable increase in T2D-DN patients. Also, we identified several significant changed T cells, B cell, and monocyte subgroups in the early-stage DN associated with several potential biomarkers for developing DN, such as CTLA-4, CXCR3, PD-1, CD39, CCR4, and HLA-DR. However, these potential biomarkers require further validation in a second cohort on a more extensive series of patients.

In the current study, the major limitations are small sample numbers and neither based on the prospective study nor further validation in the second cohort. In addition, there exists an age difference between the T2DM patients with and without diabetic nephropathy. Therefore, we should further consider the risk of age in the immunological changes during the progression of DN. Moreover, we couldn’t exclude non-complicated T2DM patients who will develop end-stage DN during the disease. Furthermore, the biomarkers identified in the current research should be validated and followed up with a second visit to determine their role in the progression of DN. In addition, although most T2DM patients only received oral hypoglycemic drugs or insulin, it’s challenging to guarantee whether these drugs affect the host immune system. Nevertheless, compared with the complicated drug intake of patients with end-stage DN, the immune cell characteristics in the PBMC of T2DM patients with early-stage DN could more genuinely reflect the immune status of disease progression.

## Data availability statement

The original contributions presented in the study are included in the article/**Supplementary Materials**. Further inquiries can be directed to the corresponding authors.

## Author contributions

Conception and design of the research: QH. Acquisition of data: LW, YL, and WH. Analysis and interpretation of data: JJ. Statistical analysis: DZ. Funding support: JJ, YN, and QH. Drafting the manuscript: JJ. Revision of manuscript for important intellectual content: YN. All authors have reviewed and proved the final manuscript.
