# Corrigendum: Depiction of immune heterogeneity of peripheral blood from patients with type II diabetic nephropathy based on mass cytometry

**DOI:** 10.3389/fendo.2023.1168889

**Published:** 2023-03-07

**Authors:** Juan Jin, Longqiang Wang, Yongjun Liu, Wenfang He, Danna Zheng, Yinhua Ni, Qiang He

**Affiliations:** ^1^ Urology & Nephrology Center, Department of Nephrology, Zhejiang Provincial People’s Hospital, Affiliated People’s Hospital, Hangzhou Medical College, Hangzhou, Zhejiang, China; ^2^ Department of Thyroid and Breast Surgery, The Central Hospital of Wuhan, Tongji Medical College, Huazhong University of Science and Technology, Wuhan, China; ^3^ College of Biotechnology and Bioengineering, Zhejiang University of Technology, Hangzhou, China; ^4^ Department of Nephrology, The First Affiliated Hospital of Zhejiang Chinese Medical University (Zhejiang Provincial Hospital of Traditional Chinese Medicine), Hangzhou, Zhejiang, China

**Keywords:** high-dimensional mass cytometry, diabetic nephropathy, immune disorder, peripheral blood mononuclear cell (PBMC), type II diabetes mellitus

In the published article, there was an error in **Figure 1** as published. In **Figure 1B** the legend of the data for T2D and T2D-DN groups were erroneously the same. The corrected **Figure 1** and its caption appear below.

**Figure 1:**
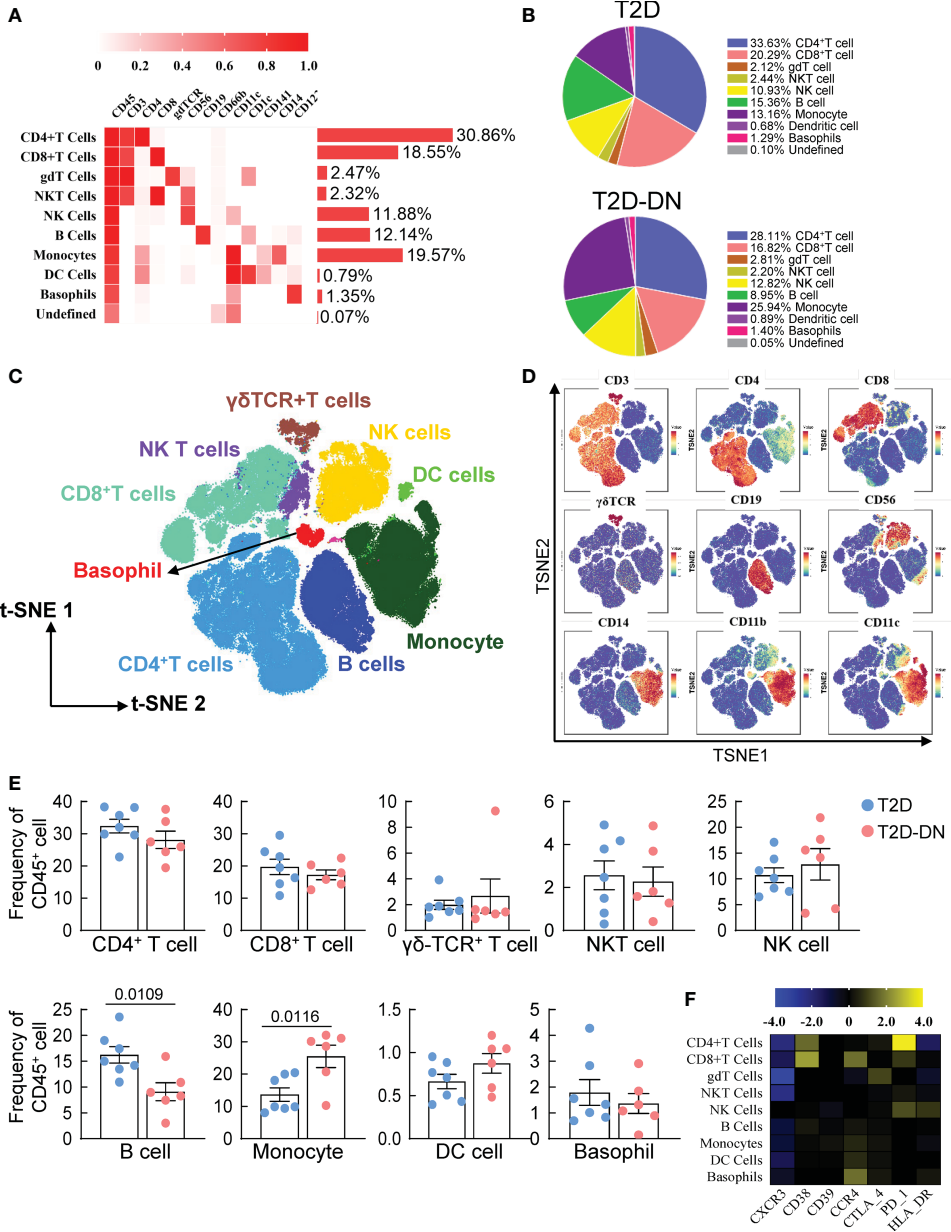
Peripheral immunity signature traits in the early-stage diabetic nephropathy patients. **(A)** Heatmap analysis for the overall proportions of major immune cell subsets in diabetic nephropathy **(B)** Pie chart characterizing the differences of major immune cell subsets proportions between T2D-DN and T2D patients. **(C)** Distributions of major immune cell subsets **(D)** The key immune cell markers for immune cell subsets are analyzed by t-SNE algorithm. **(E)** Statistical frequency differences of immune cell subsets between T2D-DN and T2D patients. **(F)** Heatmap analysis for the expressions of functional immune cell markers in the measurable immune cell subsets. Data are expressed as means ± SEM, n = 6 in T2D-DN group and n = 7 in T2D group.

The authors apologize for this error and state that this does not change the scientific conclusions of the article in any way. The original article has been updated.

