# Supplementary material for: Depiction of immune heterogeneity of peripheral blood from patients with type II diabetic nephropathy based on mass cytometry
Source: Front Endocrinol (Lausanne). 2023 Jan 6;13:1018608. doi: 10.3389/fendo.2022.1018608 (PMC9853014; doi:10.3389/fendo.2022.1018608)
Supplement: Supplementary file 1 [file DataSheet_1.docx]

Table S1. The antibody reagents used in CyTOF

| Protein | Clone | Lot Number | Metal isotope | | Surface (S) / Intracellular (I) staining | Company |
| --- | --- | --- | --- | --- | --- | --- |
| CD45 | HI30 | 304002 | | 89Y | S | BioLegend |
| CD3 | UCHT1 | BE0231 | | 115In | S | Bio Cell |
| CD68 | Y1/82A | 333802 | | 139La | I | BioLegend |
| CD56 | NCAM16.2 | 559043 | | 141Pr | S | BD |
| γδTCR | 5A6.E9 | PLLTECH | | 142Nd | S | PLLTECH |
| CD196(CCR6) | G034E3 | 353402 | | 143Nd | S | BioLegend |
| CD14 | M5E2 | 301810 | | 144Nd | S | BioLegend |
| CD45RA | HI100 | 304102 | | 145Nd | S | BioLegend |
| CD123(IL-3Ra) | 6H6 | 306002 | | 146Nd | S | BioLegend |
| CD183(CXCR3) | G025H7 | 353750 | | 147Sm | S | BioLegend |
| CD19 | HIB19 | 302214 | | 148Nd | S | BioLegend |
| CD169(siglec-9) | 7-239 | 346002 | | 149Sm | S | BioLegend |
| CD1c | L161 | 331502 | | 150Nd | S | BioLegend |
| CD38 | HIT2 | 303502 | | 151Eu | S | BioLegend |
| CD195(CCR5) | J418F1 | 359102 | | 152Sm | S | BioLegend |
| CD303(BDCA2) | 201A | 354215 | | 153Eu | S | Biolegend |
| CD163 | GHI/61 | 333602 | | 154Sm | S | BioLegend |
| CD206(MMR) | 15-2 | 321112 | | 155Gd | S | BioLegend |
| CD204(SR-AI) | 351615 | MAB2708 | | 156Gd | S | RD |
| CD39 | A1 | 328202 | | 157Gd | S | BioLegend |
| CD194(CCR4) | L291H4 | 359402 | | 158Gd | S | BioLegend |
| CD11c | BU15 | 337202 | | 159Tb | S | BioLegend |
| CD25 | 24212 | MAB1020 | | 160Gd | S | RD |
| CD152(CTLA-4) | 14D3 | 14-1529-82 | | 161dy | I | eB |
| FoxP3 | PCH101 | 14-4776-82 | | 162Dy | I | eB |
| CD159a(NKG2A) | 131411 | MAB1059 | | 163Dy | S | RD |

Table S1 (Continued)

| Protein | Clone | Lot Number | Metal isotope | Surface (S) /Intracellular (I) staining | Company |
| --- | --- | --- | --- | --- | --- |
| CD141 | M80 | 344102 | 164Dy | S | BioLegend |
| CD66b | G10F5 | 305102 | 165Ho | S | BioLegend |
| Perforin | B-D48 | ab47225 | 166Er | I | Abcam |
| CD197(CCR7) | G043H7 | 353222 | 167Er | S | BioLegend |
| T-bet | 4B10 | 644802 | 168Er | I | BioLegend |
| CD314(NKG2D) | 1D11 | 320814 | 169Tm | S | BioLegend |
| CD127(IL-7Ra) | A019D5 | 351302 | 170Er | S | BioLegend |
| GATA3 | TWAJ | 14-9966-82 | 171Yb | I | eB |
| RORγt | 600214 | MAB6109 | 172Yb | I | RD |
| GranzymeB | GB-11 | 3173006B | 173Y | I | fluidigm |
| CD279(PD-1) | EH12.2H7 | 329926 | 174Yb | S | BioLegend |
| CD16 | 3G8 | 302014 | 175Lu | S | BioLegend |
| HLA-DR | L243 | 307612/307648 | 176Yb | S | BioLegend |
| CD4 | RPA-T4 | 300516 | 197Au | S | BioLegend |
| CD8 | RPA-T8 | 301018/301074 | 198Pt | S | BioLegend |
| CD11b | M1/70 | 101202 | 209Bi | S | BioLegend |
